# Draft Genome Sequences of Coral-Associated Bacterium *Hoeflea* sp. Strain E7-10 and Dinoflagellate-Associated Bacterium Hoeflea prorocentri PM5-8

**DOI:** 10.1128/mra.00018-23

**Published:** 2023-04-04

**Authors:** Guoqiang Zhang, Qiaoyan Wei, Zongyao Zhang, Lin Cai

**Affiliations:** a School of Life and Environmental Science, Guilin University of Electronic Technology, Guilin, Guangxi, People’s Republic of China; b Shenzhen Institute of Guangdong Ocean University, Shenzhen, Guangdong, People’s Republic of China; Montana State University

## Abstract

Here, we report the draft genome sequences of *Hoeflea* sp. strain E7-10 and Hoeflea prorocentri PM5-8, isolated from a bleached hard coral and a culture of marine dinoflagellate, respectively. Genome sequencing for host-associated isolates *Hoeflea* sp. E7-10 and *H. prorocentri* PM5-8 can provide basic genetic information to explore potential roles in their hosts.

## ANNOUNCEMENT

*Hoeflea* is a genus of Gram-negative, aerobic, rod-shaped, and non-spore-forming bacteria that belongs to the phylum *Proteobacteria*, the class *Alphaproteobacteria*, the order *Rhizobiales*, and the family *Phyllobacteriaceae*. Members of *Hoeflea* were mainly isolated from marine environments such as seawater, sediments, dinoflagellates, and corals (1, 2). Here, we isolated *Hoeflea* sp. strain E7-10 from a bleached hard coral, Porites lutea, collected in Daya Bay, Guangdong, China. A small coral fragment was ground to slurry with 0.85% NaCl. Serial dilutions (10^0^ to 10^−2^) were made, and 0.1 mL of each dilution was plated on marine nutrient agar at 25°C for 4 weeks. The obtained isolates were picked, purified, identified, and stored for further study. Hoeflea prorocentri PM5-8 was isolated by Zhejiang Ocean University from the cell culture of a marine dinoflagellate, Prorocentrum mexicanum PM01, collected in southern Taiwan, China (3). A query based on a nearly full-length 16S rRNA gene (27F and 1492R) of *Hoeflea* sp. E7-10 using the EzBioCloud server (4) revealed that its closest relative was *H. prorocentri* PM5-8, showing 98.01% similarity. *H. prorocentri* PM5-8 was purchased from the China Center for Type Culture Collection (CCTCC) for genome sequencing and comparison. Both *Hoeflea* sp. E7-10 and *H. prorocentri* PM5-8 were cultured on marine agar/broth 2216 (BD Difco, USA) at 28°C.

The genomic DNA of *Hoeflea* sp. E7-10 and *H. prorocentri* PM5-8 was extracted using the SteadyPure bacterial genomic DNA extraction kit (AG, China). Sequencing libraries were prepared with the Nextera XT DNA library preparation kit (Illumina, USA). Genome sequencing of *Hoeflea* sp. E7-10 and *H. prorocentri* PM5-8 was conducted by Novogene Co., Ltd. using the Illumina NovaSeq 6000 system with a paired-end (PE) mode of 2 by 150 bp. Shotgun PE reads were cleaned with the NGS QC toolkit v2.3.3 (5) and assembled *de novo* to obtain draft genomes by SPAdes 3.15.3 (6, 7). Draft genomes were annotated through the NCBI Prokaryotic Genome Annotation Pipeline (PGAP) 6.3 (8). The completeness and quality of genomes were evaluated by CheckM 1.1.3 (9). The average nucleotide identity (ANI) was calculated with ANI Calculator (10). Default parameters were used for all software tools unless otherwise specified.

After removal of low-quality reads, 1.74 Gb of PE reads of *Hoeflea* sp. E7-10 was obtained for genome *de novo* assembly. The draft genome sequence of *Hoeflea* sp. E7-10 consisted of 54 contigs with a genome size of 5.58 Mb and over 300-fold genome coverage, an *N*_50_ value of 277,445 bp, and an average GC content of 60.27%. The genome contains 5,217 coding sequences and 53 RNA genes (45 tRNA, 4 rRNA, and 4 noncoding RNA [ncRNA] genes). After filtration of low-quality reads, 1.55 Gb of PE reads of *H. prorocentri* PM5-8 was obtained for genome *de novo* assembly. The draft genome sequence of *H. prorocentri* PM5-8 consisted of 2 contigs with a genome size of 5.04 Mb and over 300-fold genome coverage, an *N*_50_ value of 4,582,609 bp, and an average GC content of 58.09%. The genome contains 4,653 coding sequences and 50 RNA genes (43 tRNA, 3 rRNA, and 4 noncoding RNA [ncRNA] genes). The completeness of *Hoeflea* sp. E7-10 and *H. prorocentri* PM5-8 genomes was 100% for both, and the genome contamination was 0.97% for both. The ANI between *Hoeflea* sp. E7-10 and *H. prorocentri* PM5-8 genomes was 75.95%.

### Data availability.

The draft genome sequences of *Hoeflea* sp. E7-10 and *H. prorocentri* PM5-8 have been deposited at DDBJ/EMBL/GenBank under accession numbers JAPJZH000000000 and JAPJZI000000000, respectively. The genome sequencing raw reads of *Hoeflea* sp. E7-10 and *H. prorocentri* PM5-8 have been deposited in the NCBI Sequence Read Archive (SRA) under accession numbers SRR22307417 and SRR22307416, respectively.
